# Editorial: Red Blood Cell Vascular Adhesion and Deformability, Volume II

**DOI:** 10.3389/fphys.2022.849608

**Published:** 2022-02-18

**Authors:** Helene Guizouarn, Gregory Barshtein

**Affiliations:** ^1^Institut de Biologie Valrose, Université Côte d’Azur, Nice, France; ^2^Biochemical Department, The Faculty of Medicine, Hebrew University of Jerusalem, Jerusalem, Israel

**Keywords:** red-blood-cell (RBC), RBC deformability (ED), RBC adhesion, sickle anemia, thalassemia, diabetes, blood viscosity

Red blood cells (RBCs) are highly specialized cells that carry respiratory gases (McMahon et al.). During circulation, erythrocytes are subjected to shear stress caused by blood flow and extreme physical restrictions, especially when pushed through the narrow capillaries and sinuses of the spleen (Yedgar et al., 2002; Li et al., 2021; Van Cromvoirt et al., 2021). RBC has unique flow-affecting properties that play a major role in hemodynamics, and their normal function is essential for adequate blood flow and tissue perfusion in large and small blood vessels (Yedgar et al., 2002; Barshtein et al., 2018b). These features include the deformability of RBCs (Lu M. et al.; Atwell et al.; Barshtein et al.; Hsu et al.) and their ability to adhere (Lu M. et al.) to the endothelial cells lining the vascular system. Under normal conditions, RBCs are entirely deformable, and their adhesion is negligible (Yedgar et al., 2002; Barshtein et al., 2018b). However, under numerous pathological conditions (Lu M. et al.; Atwell et al.; Hsu et al.) and during cold storage in the blood bank (Barshtein et al.), the rigidity and adhesion of RBCs increase, which contributes to impaired flow behavior.

The deformability of RBCs is predominantly modulated by the erythrocyte shape, its membrane structure/composition (Barshtein et al.; Lu D. et al.; Hsu et al.), and the cytosol content (McMahon et al.). Herewith, RBC adhesion to endothelial cells (EC) depends on external membrane composition (Koshkaryev et al., 2009; Colin et al., 2014) and extracellular medium content (Barshtein et al.).

This research topic contains articles devoted to biophysical (Barshtein et al.; Lu M. et al.; Atwell et al.), biochemical (Lu D. et al.; McMahon et al.), and physiological (McMahon et al.; Lu D. et al.; Atwell et al.; Hsu et al.; Sun et al.) aspects of RBC flow-affecting properties, as well as the role of these features in blood microcirculation. In addition, the effect of various pathologies (Lu M. et al.; Atwell et al.; Sun et al.) and *in-vitro* aging (Barshtein et al.) on the features under consideration are discussed here. The authors pay special attention to the deformability and adhesion of RBC characteristics for two types of hemoglobinopathies; sickle anemia (Lu M. et al.; Lu D. et al.) and thalassemia (Lu D. et al.).

## Role of Intracellular ATP in RBC Functionality

Adenosine triphosphate (ATP) is a major player as a signaling molecule in blood microcirculation (McMahon et al.). It is released by erythrocytes when subjected to solid shear stresses (McMahon et al.), large enough to induce a sufficient shape deformation (Mcmahon, 2019). RBCs produce ATP from the anaerobic conversion of D-glucose to lactate. Alternatively, RBCs can make 2,3-biphosphoglycerate (2,3-BPG, or 2,3-DPG) that reduces the hemoglobin affinity to oxygen (McMahon et al.). Most ATP is used to maintain the ion balance, cell volume, and RBC deformability (Mcmahon, 2019). And although there are conflicting data (Barshtein et al.) on the relationship between intracellular ATP content and RBC deformability, the effectiveness of restoring the ATP level in packed RBCs for reducing cells stiffness has been demonstrated previously (Barshtein et al., 2018a).

## Role of Phosphatidylserine (PS) Externalization on RBC Functionality

PS in normal RBCs is restricted to the inner leaflet of the membrane bilayer, but it is externalized in a high but variable proportion of pathological or aged cells (Kuypers and De Jong, 2004). PS externalization can be stimulated during cell aging (Barshtein et al.), external influence, or as a consequence of pathology (Lu D. et al.). In this issue, Lu D. et al. conclude that PS exposure in RBCs from sickle anemia patients increases under hypoxic, acidic, and hypertonic conditions but is markedly reduced by high urea concentrations thus, showing the role of the renal medulla in RBC sickling. The externalization of PS on the surface of the cell membrane, on the one hand, affects RBC functionality, and on the other hand, is a marker for cell aging (Kuypers and De Jong, 2004). Moreover, the relationship between PS extracellular level and RBC adhesion to the endothelium has previously been demonstrated for some RBC pathologies (sickle anemia, thalassemia, malaria, etc.) (Wautier and Wautier, 2013, 2020; Fraser et al., 2021) and cold storage (Barshtein et al.)

## Flow-Affecting Properties of RBC in Health and Diseases

RBC biophysical properties contribute to blood flow features. Impairment of these flow-affecting properties of RBCs was associated with RBCs pathology, a characteristic of numerous diseases, such as sickle cell anemia (Lu M. et al.; Lu D. et al.), thalassemia (Lu D. et al.), cerebral malaria (Wautier and Wautier, 2013), sepsis (Mcmahon, 2019), and diabetes (Sun et al.), in which, microcirculation disorders are an integral feature of their pathophysiology (Yedgar et al., 2002). The development of microfluidic devices that can concurrently measure RBC deformability and adhesiveness (Lu M. et al.) or enable motion study of RBC (Atwell et al.) are proposed to discriminate pathological RBC. These devices should fulfill the need for biomedical markers to identify abnormal RBCs.

Unfortunately, the ability to improve the flow-affecting properties of RBCs with the help of medicaments is limited; however, the targeted application of the dietary approach and nutritional supplements can significantly improve the situation (Stupin et al., 2019). Information on the effect of physical activity on the flow-affecting properties of erythrocytes is controversial. The outcome is primarily determined by the exercise protocol and the physiological state of the subjects (Connes et al., 2011; Mury et al., 2017; Nader et al., 2019). So, Hsu et al. showed that supervised cycling training improves functional aerobic capacity, enhances erythrocyte membrane stability as well as osmotic deformability, and consequently promotes peripheral arterial disease patients.

## Deformability of Stored RBCs

The effect of storage on RBCs deformability has a long history and began in the mid-1980s. At present, with the advent of new approaches (microfluidics) which enable to determine the distribution of deformability in the cell population (Relevy et al., 2008; Guruprasad et al., 2019; Barshtein et al., 2020b; Islamzada et al., 2020; Man et al., 2020), the scientific interest in this field has increased. Most researchers observed a significant fall in cell deformability after 14–21 days of storage (Barshtein et al.). At the same time (for several units), the stability of deformability was observed throughout storage (Barshtein et al.). There was also significant variability in the deformability of the RBCs, both between different donors and between RBC units. In addition, it was found that the preparation of the RBC unit causes an alteration in the deformability of cells (Barshtein et al., 2020a).

## Conclusion

We hope that this Research Topic (which is a continuation of the previous collection on the same issue), which focuses on the flow-affecting properties of RBCs, will contribute to the knowledge about RBCs. We thank all the participants in the collection who presented their exciting publications. We also hope that the topic we have raised will attract many new researchers in future.

## Author Contributions

HG and GB wrote the manuscript. All authors listed have made a substantial, direct, and intellectual contribution to the work and approved it for publication.

## Conflict of Interest

The authors declare that the research was conducted in the absence of any commercial or financial relationships that could be construed as a potential conflict of interest.

## Publisher's Note

All claims expressed in this article are solely those of the authors and do not necessarily represent those of their affiliated organizations, or those of the publisher, the editors and the reviewers. Any product that may be evaluated in this article, or claim that may be made by its manufacturer, is not guaranteed or endorsed by the publisher.
